# Perceptions of undergraduate nursing students toward providing care to COVID-19 patients

**DOI:** 10.3389/fpubh.2022.954907

**Published:** 2022-09-29

**Authors:** Fakhrudin Faizi, Seyed Tayeb Moradian

**Affiliations:** Atherosclerosis Research Center and Nursing Faculty, Baqiyatallah University of Medical Sciences, Tehran, Iran

**Keywords:** nursing student, perception, experience, COVID-19, posttraumatic growth

## Abstract

**Background:**

Undergraduate nursing students were inevitably recruited to provide care in response to overloaded hospitals with COVID-19 patients. The placement was potentially a stressful event and we aimed to understand the students' perception of direct nursing care during the pandemic.

**Methods:**

This qualitative content analysis study carried out in 2020 (May-June) in Tehran, I.R. Iran. Using explanatory questions, we interviewed 15 undergraduate nursing students who provided direct care for at least 2 months to patients hospitalized with COVID-19 in the beginning of the pandemic to obtain their deep experiences. We applied the MAXQDA 10 to extract codes, used the qualitative content analysis method for investigation, and then applied the Lincoln and Guba criteria for rigor and trustworthiness. The study was approved by the National Committee for Ethics in Biomedical Research (IR.BMSU.REC.1399.070. available at: https://ethics.research.ac.ir).

**Results:**

Four hundred and 54 codes were identified, which were then sorted into 12 categories underlying four main themes of “hard and unpredictable,” “posttraumatic growth,” “on the path to self-determination,” and “commitment.”

**Conclusion:**

Even though the undergraduate nursing students experienced some stress in the beginning, with close support, their caregiving skills improved and they were able to meet the national healthcare needs. More studies are needed to confirm our findings regarding the experiences of the nursing students in combating COVID-19.

## Introduction

The novel coronavirus (COVID-19) pandemic exacerbated a global nursing shortage concern that healthcare provision is being compromised due to a lack of qualified nurses (1). For years, the shortage was a “major challenge of [the] health system” in Iran, the second country that was affected by the coronavirus (2–4). Iran also was one of the top ten countries considering COVID-19 mortality (5, 6) due to the fact that 138 healthcare workers lost their lives due to coronavirus by 23 July 2020 (7).

To meet the needs, Iran's Ministry of Health and Medical Education (MoHME) inevitably drafted under-graduate nursing students to care for patients with COVID-19 in response to hospitals receiving COVID-19 cases (8). Baqiyatallah Hospital was the first hospital in the country to receive cases of COVID-19 in February 2019 (9). Urgently, the Faculty of Nursing in Baqiyatallah University of Medical Sciences (BUMS) formed an executive committee and all concerns and healthcare needs were considered. Following the Action Plan, the students were recalled and allocated to the hospital wards under the supervision of their educators. Even though they returned voluntarily, the decision could have disturbed them as being away from their families may have triggered some psychological concerns (10, 11). The students didn't have any experience in caring for people with COVID-19 and even experienced nurses also needed psychological support during the pandemic situation (12, 13).

Beyond the pandemic, undergraduate nursing students are vulnerable, and the clinical setting usually has a stressful influence on nursing students, as has been well-documented in previous studies. Al Rasheed et al. (14) study showed that the students from a college of nursing have had the highest level of stress among public health university students while the average stress in whole university students was moderate (14). Ahmed et al. (15) also reported that nursing students have experienced a “moderate” degree of stress in clinical practice and the students frequently used problem-solving and avoidance strategies for tackling the stress (15). To date, few documents have reported undergraduate nursing students' perceptions of direct care during the COVID-19 pandemic (16). We aimed to hear their experiences of caring for the patients and then focus on their main concerns to help guide health policy makers and managers to better handle similar crises in the future.

## Materials and methods

The qualitative content analysis study carried out on May-June in 2020 in Tehran, Iran, to illuminate the experience of the students of care provided to COVID-19 patients.

### Participants

Eighty-four students participated in a preliminary short course outlining “how to provide care to the patients” prior to embarking. After 2 months using purposive sampling, 15 participants (10 males) including 10 nursing students, 3 anesthesiologists, and 2 operation room technologists were interviewed. Sampling continued until data saturation was obtained. Sample of follow up questions were: “Tell us about your feelings when you were introduced into the hospital wards,” “How did you spend your time during the pandemic?”, “What was it like to care for a patient with COVID-19” and “What did you think when you decided to go back to the hospital?”. For enhancing the maximum variation, students from different disciplines and semesters including both male and female were interviewed. Those who had at least 2 months of direct caring from COVID-19 patients' experience, were invited to participate in the study.

### Data gathering

Semi-structured interviews lasting 25–45 min were carried out. After several warmup open-ended questions, the interviews were continued with probing and explanatory questions. Students were asked to narrate their experience in as much detail as possible. Interviews were conducted in the faculty of nursing office in favor of the students. All 15 interviews were done in the Persian language by a single interviewer. Only the quotations that are presented in the findings section were translated into English.

### Data analysis

The Graneheim and Lundman approach for qualitative content analysis was used (17). After each interview, it was transcribed verbatim. First the text was read several times to obtain a general sense and then meaning units were extracted and coded. Based on similarities and differences, they were organized in categories and subcategories. Using a constant comparison method, latent and underlying meaning were extracted as themes. The emerged themes then were reviewed together with another colleague (F.F). The MAXQDA ver.10 software was used for data analysis and the Lincoln and Guba criteria were followed for rigor and trustworthiness. The four-dimensional criteria (credibility, dependability, confirmability, and transferability) have been proposed to strengthen the robustness and rigor of qualitative studies in emergency departments. Considering the emergent situation that we faced at the beginning of the pandemic (nursing shortage and extensive healthcare needs) the strategies proposed by Lincoln and Guba were followed point by point through close supervision in the field, multiple interviews, peer checking, detail in describing the research method, comparing our data with other similar experiences within the context, purposeful sampling, and data saturation (18).

### Ethical considerations

We received the ethical approval number of IR.BMSU.REC.1399.070 from the National Committee on Biomedical Research. The research objectives were explained to the participants and written informed consent was received. The participants' permission was also obtained for recording their interviews. The time and place of the interviews were determined by participants, and they were free to stop if they felt exhausted or distressed. Having no tendency to continue, disagreements to record their voice, and or take their notes were used as exclusion criteria. One of the female students didn't agree to voice recording, so the interview was done using simultaneous note taking.

## Findings

**Figure 1:**
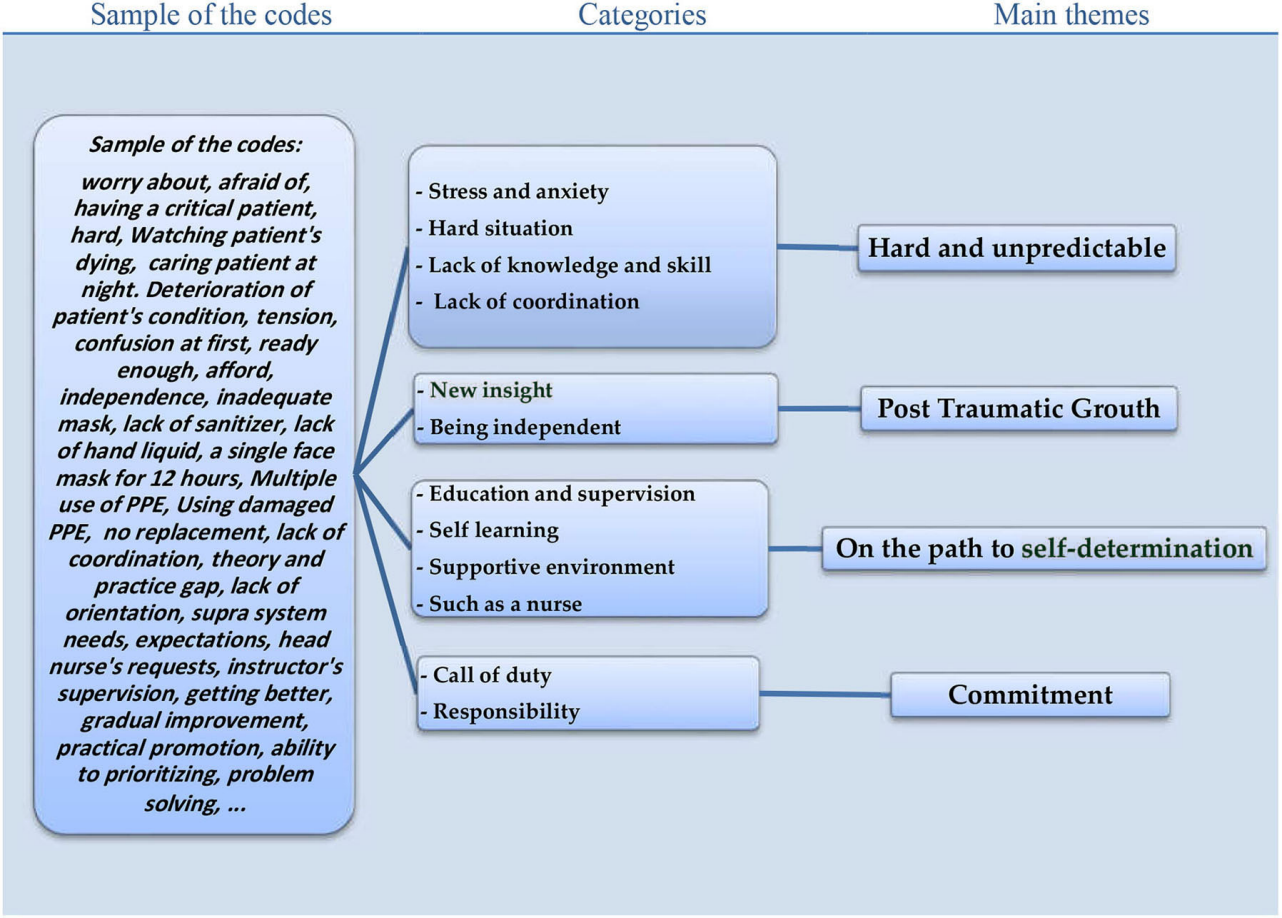
Qualitative conventional content analysis of the students' experience in providing care for patients with COVID-19 during the pandemic.

Data analysis revealed 454 codes that were sorted into 12 categories outlining four main themes including “hard and unpredictable,” “on the path to self-determination,” “posttraumatic growth,” and “commitment” (Figure 1).

### Hard and unpredictable

This theme comprised of categories of “stress and anxiety,” “hard situation,” “lack of knowledge and skill,” “lack of personal protective equipment” (PPE), and “lack of coordination.”

#### Stress and anxiety

Students experienced stress for two reasons: The main cause of stress was related to the nature of the disease. In addition to worrying about becoming infected, they were concerned about contagion to the family, colleagues, students, and immunocompromised patients. The second source of stress was their knowledge and skill deficiency and a fear of harming patients.

A female nursing student said “*I was not worried about myself at all. Patients' immunity was poor. I was afraid of passing contagion to them.”*

#### Hard situation

The hard situation had different sources, including lack of PPEs, nursing shortage and their fatigue, crowding, families request for coming back home, and being away from family and friends. Also, they were under pressure due to patients' lack of response to treatment, being critical, and in some cases death.

A female nursing student said “*Having a critical patient was very hard for me, especially the night that the patient died. He was fine at 9 o'clock at night. Within 10 min he became critical. I was very much stressed.”*

#### Lack of knowledge and skill

Students reported their knowledge, skills, and readiness for clinical practice in different aspects including documentation, using PPE, therapeutic intervention, and drug interactions.

A male student stated “*The first day that the patient was handed over to me, I was confused. I think I wasn't ready enough to care independently*.”

In the first days, PPE was not adequate. With the passage of time, the supply chain improved and PPE was adequately available. Also in regard to the supply chain, different wards were not allocated based on their needs.

A male student stated “*In the first days of the pandemic, the number of protective equipment was inadequate. We had to use a single mask for a 12-h shift. If it was damaged, no replacement would be made.”*

#### Lack of coordination

One of the main problems was the lack of coordination between nursing faculty and the hospital. Students' duties, allocation to the hospital wards, the instructor's role, unclear expectation from students, and inadequate attendance of the instructors were the main problems.

A male student said “*At first, the coordination between nursing faculty and the hospital was not good. The head nurse had some expectations that were discordant with our instructor.”*

### Post traumatic growth

Despite the hard situation of working in infected wards, students gained worthwhile experiences and skills. Their independence was improved. They were working as educated nurses. Of course, this independence was not complete, and they needed education, support, and supervision.

#### New insight

Students were not ready for working overtime and they had to work independently. Consequently, they gained new experiences and skills. They learned to be responsible. Their judgment about the patient's condition improved. Documentation, decision making, using their previous knowledge, teamwork, relationship with patients and colleagues, self-esteem, working in an infected ward, and readiness for catastrophic situations were some of their newly acquired skills and capabilities. They saw themselves as better and more patient than before. Clinical skills were significantly improved, and stress repetition decreased.

A female student said “*Day by day my skills improved. By spending time, I felt that I can afford myself. I can prioritize the problems and then one by one solve them”*

#### Being independent

This was the first time that students had to work independently. They had a path of being independent. During the students' regular shifts, they had no accountability, and the main responsibility was for their instructors and patients' nurses, so they referred to their instructors as soon as they faced the slightest challenge. In the new situation, they had to support the patient independently. This experience was before their graduation and helped them to be ready for graduation. They were able to use their experience in the future and manage their patients easily. Also, they were able to manage their patients during the night shifts independently and felt useful because of this independence.

A male student said “*Over time, I was able to prove [to] myself that I can work as a completely independent nurse. I could easily manage my patients in the night shifts. I felt myself to be more useful.”*

### On the path to self-determination

Students had to do some work being dependent due to inadequate knowledge and skill. They were gradually trained and supervised so that they could work as independent nurses. Additionally, the supportive environment and some strategies used by students helped them.

#### Education and supervision

Wherever an individual has to work independently, they must first be trained and then monitored. First, the student nurses were oriented for several days, and then they worked with a nurse educator or an experienced nurse. They observed the behavior and the work was explained to them, and then feedback was received. In the early days, students worked functionally and with their supervising nurses. Afterward, they provided care for the patients independently, but under the supervision of the head nurse or an experienced nurse. Procedures were monitored continuously and reviewed. A male student said “*Head nurse referred me to the nurse educator. He told me: Observe anything and report to me your notes. In [the] following days, he taught me [the] required procedure during implementation.”*

#### Self-learning

Students had strategies for learning such as observation and experience, asking the head nurse, studying, learning from others, and using the supervision of skilled nurses. They were checking the errors and ambiguous points with head nurses. Most of the students had a checklist for their daily duties too.

A female nurse stated “*I was writing everything in the ward. I checked them at the end of shifts with [the] head nurse or other experienced nurses. I had a checklist in my box. Every hour I was checking for missed work*.”

#### Supportive environment

The students considered the supportive environment to be the most important thing in dealing with this critical situation and its difficulties. The nurses had a good relationship with the students and were replying to their questions. Nurses supported students when they did not have enough information or had critically ill patients. Experienced nurses provided emotional and psychological support. Also, in the case of attendance of instructors, they were supported.

A female student said: “*Head nurse was quite supportive. This support resulted in higher self-esteem. Also, other colleagues were supportive. One night when my patient turned critical, my colleague accepted the responsibility of the patient*.”

#### Such as a nurse

After achieving independence, the students were present in their shifts as staff. They cared for the patients as nurses, and they felt good to be seen. They acknowledged that they were respected and not abused.

A male student said: “*After several orientation shifts, I was independent. I was respected like a nurse, and they expected me to do the duties completely.”*

### Commitment

The students attended voluntarily and somehow considered it as their duty to attend. This theme has two categories of “call of duty” and “responsibility.”

#### Call of duty

Students said that COVID-19 patients are just like any other patients and have the right to receive medical care. They felt it was their duty to help during the shortage of nurses, and while the nurses were fatigued. They felt good providing psychological support to the patients and felt that they had to be strong so that the patients would be encouraged to see them. It was like a battlefield for them, and they took it as a duty to be present on this battlefield.

A male student said: “*We prayed for the assistance. This was our mission because our people needed help. So, I felt a duty to attend in this field.”*

#### Responsibility

Students were sensitive to doing things right. They were trying to be highly accurate in caring for patients. They were monitoring continuously, because taking responsibility for patients' health was difficult and stressful for them. They followed all health protocols while working.

A female student said: “*I was working with cancer patients. Their immunity was compromised. I was washing my hands before and after every procedure. In case of a patient having leukopenia, I even washed the devices such as blood pressure cuff using an alcohol pad*.”

## Discussion

To date, limited data are available about experiences of undergraduate nursing students providing care for patients with COVID-19. However, similar results were recently reported by Rahmani et al. (16) about experiences of nursing students that provided care for Covid-19 patients concluding the main categories of “conflicting emotions, psychosocial problems, self-awareness, and empowerment” (16). Beyond the comparable consequences, they reported only the experiences of student nurses. As mentioned previously, Baqiyatallah hospital was the first one in the country that received cases of COVID-19 patients imposing a contingent circumstance to all the nursing students and their families.

Considering the theme of “*commitment*,” Lovic et al. (19) from Croatia reported that the nursing students worried about their families' health, but recognized their responsibility to the community and risks of being a nurse (19). They did not mention if the students were faced with a lack of PPEs, medical shortages, sanitizer etc. or the obligation by their governments' back to work mandates that the students came across in our study.

Considering the theme of “*hard and unpredictable situation*,” Velarde et al. from Spain (2021) reported the undergraduate student nurses experienced fear of possible contagion to their cohabitant, stress, insomnia, nightmares, and anxiety. They concluded the students had emotional tension during clinical education that was eased by support of co-workers and their families recommending psychological support in probable upcoming situations (20). The study did not articulate if the students provided direct care to patients with COVID-19.

Haggard's study from Arkansas (2022) on undergraduate nursing students' health behaviors showed the students experienced more panic attacks, headache, and more consumption of alcohol prior to the pandemic (quarantine period) (21). It was not understood whether the students were directly involved in COVID-19 patient care.

On medical education during the pandemic however, Khamis et al. (22) expressed alarm about the risk of the recommendation by the Association of American Medical Colleges and the Liaison Committee on Medical Education (LCME) that medical students should spend two weeks in an emergency department during the pandemic (22); however, Long et al. (4) at Penn State College of Medicine, concluded that the COVID-19 pandemic provided a “unique opportunity” to increase medical students' competencies (4). Similarly, the themes of “*on the path to self-determination*” and “*commitment*” in our study showed the students have considered the pandemic as an inevitable but unique condition in their career.

Considering the theme of ‘*hard and unpredictable*,” Fan et al. (23) interviewed interdisciplinary nurses (NTs with no experience in caring for patients with infectious disease) and found similar themes of “unfamiliar work content” and “ambiguous roles” but discordantly they reported that NTs experienced three times more stress than the nurses with previous experience, meanwhile the majority of the NTs (19 out of 26) had more than 6 years of work experience (23). Ulenaers et al. (24) also reported the experiences of the nursing students in practice during the pandemic that they were worried, received less support, had less learning situations, and had doubts about being a nurse (24).

The study reflects “sounds and feelings” of even a small group of undergraduate nursing students' perceptions that were legitimately faced with such a hard situation. The findings may help nursing managers and healthcare authorities in planning for similar crises in the future. As a limitation, the study was single-centered, and it was not possible to interview with similar students from other universities because of the restrictions during the pandemic.

## Conclusion

The study revealed that although caring for COVID-19 patients was a very stressful situation for undergraduate nursing students, with supervision and support in the clinical setting, they found the catastrophe as a way to develop their competencies to meet the healthcare needs. Authorities may consider the undergraduate students as a “hidden arm” to help meet healthcare needs in the future. More studies can better describe their experiences under stressful conditions.

## Data availability statement

The original contributions presented in the study are included in the article/supplementary material, further inquiries can be directed to the corresponding author.

## Ethics statement

The studies involving human participants were reviewed and approved by National Committee for Ethics in Biomedical Research (IR.BMSU.REC.1399.070. available at: https://ethics.research.ac.ir). The patients/participants provided their written informed consent to participate in this study.

## Author contributions

FF and SM worked together equally during the project and drafted the manuscript. All authors contributed to the article and approved the submitted version.

## Conflict of interest

The authors declare that the research was conducted in the absence of any commercial or financial relationships that could be construed as a potential conflict of interest.

## Publisher's note

All claims expressed in this article are solely those of the authors and do not necessarily represent those of their affiliated organizations, or those of the publisher, the editors and the reviewers. Any product that may be evaluated in this article, or claim that may be made by its manufacturer, is not guaranteed or endorsed by the publisher.
